# Isolation and Characterization of 11 New Microsatellite Loci in *Erigeron breviscapus* (Asteraceae), an Important Chinese Traditional Herb

**DOI:** 10.3390/ijms12107265

**Published:** 2011-10-24

**Authors:** Xiang Li, Kexian Song, Junbo Yang, Tingshuang Yi

**Affiliations:** 1 Key laboratory of Biodiversity and Biogeography, Kunming Institute of Botany, Chinese Academy of Sciences, Kunming 650201, China; E-Mails: lixiangjohnlily@126.com (X.L.); songkexian555@yahoo.com.cn (K.S.); jbyang@mail.kib.ac.cn (J.Y.); 2 Graduate University of Chinese Academy of Sciences, Beijing 100049, China; 3 Plant Germplasm and Genomics Center, Germplasm Bank of Wild Species, Kunming Institute of Botany, Chinese Academy of Sciences, Kunming 650201, China; 4 Crop Research Institute of Sichuan Academy of Agricultural Sciences, Chengdu 610066, China

**Keywords:** *Erigeron breviscapus*, microsatellite markers, polymorphism, population genetics

## Abstract

*Erigeron breviscapus* (Vant.) Hand.-Mazz. (Asteraceae) is a species endemic to southwestern China and an important traditional Chinese herb for cardiovascular and cerebral vessel diseases. Applying a modified biotin-streptavidin capture method, 11 microsatellite loci were discovered. Polymorphism of each locus was assessed in 24 individuals collected from five wild populations. The number of alleles per locus ranged from 2 to 7, with an average of 4.273. The observed (HO) and expected (HE) heterozygosities varied from 0.250 to 0.958 and from 0.337 to 0.786, respectively. Over half of these loci were successfully amplified in two congeneric species. The developed microsatellite markers will be useful for future population genetics and conservation studies, as well as accurate identification of different varieties.

## 1. Introduction

*Erigeron breviscapus* (Vant.) Hand.-Mazz. is an important Chinese traditional herb. The whole plant is widely used to treat various diseases such as heart disease, cerebral infarction, digestive disorders and apoplexy [1]. This perennial herb is endemic to southwestern China and is found in altitudes ranging from 1200 m to 3600 m. It is mainly distributed in mid-altitude mountains and subalpine open slopes, grasslands and forest margins [2]. A series of bioactive compounds of flavonoids and phenoles have been extracted from this herb and the pharmacological effects and clinical applications of these compounds have been extensively studied [3]. This species has been widely used to make pills and injections in China [4]. More than 1000 tons of dry materials of this species are used by medical companies every year and the natural resources are becoming exhausted [5]. Therefore, population genetics studies will be quite beneficial to design conservation and management strategies for this important traditional herb. However, no microsatellite loci for *E. breviscapus* have been reported. In this study, we developed 11 microsatellite markers from *E. breviscapus*, which will be used for following studies on population genetics, conservation biology and identification.

## 2. Results and Discussion

Among the primers analyzed, 39 primers successfully amplified the target regions and 11 primer pairs were polymorphic (Table 1). Standard genetic diversity parameters, deviation from Hardy-Weinberg equilibrium (HWE) and linkage disequilibrium between pairs of loci, were estimated via GENEPOP version 4.0 [6]. The number of alleles per locus (*A*) was 2–7, with an average of 4.273. Values for observed (*H_O_*) and expected (*H_E_*) heterozygosities ranged from 0.250 to 0.958 (mean = 0.625) and from 0.337 to 0.786 (mean = 0.574), respectively. Five loci (EB-8, EB-10, EB-11, EB-28, and EB-30) deviated significantly from Hardy-Weinberg equilibrium (HWE) (*P* < 0.01). No significant linkage disequilibrium was detected between locus pairs except for locus pair EB-11 and EB-17. Congeneric species (*E. canadensis* and *E. altaicus*) amplification was further investigated using 4 individuals of each population. Half of the microsatellite loci were successfully amplified in *E. canadensis*, while eight in *E. altaicus* (Table 2).

## 3. Experimental Section

Genomic DNA was extracted from silica-gel-dried leaves using the cetyltrimethylammonium bromide (CTAB) method [7]. Then, a microsatellite enriched library was constructed using a modified biotin-streptavidin capture method [8]. Briefly, total genomic DNA (about 500–800 ng) was completely digested with MseI restriction enzyme (New England Biolabs, Beijing, China) and then ligated to MseI AFLP adaptor followed by amplification with adaptor-specific primers (5′-GATGAGTCCTGAGTAAN-3′). The amplified DNA fragments, with a size range of 200–800 bp, were hybridized to a 5′-biotinylated [(AG)_15_, (AAG)_10_ or (AC)_15_] probe. Successfully hybridized fragments were then selectively separated and captured by streptavidin-coated magnetic beads (Promega, Madison, Wisconsin, USA) [9]. Enriched fragments were amplified again with the adaptor-specific primers. PCR products were purified using PCR products purification kit (Sangon, Shanghai, China), ligated to the PGEM^®^-T vector (Promega, Madison, Wisconsin, USA), and then transformed into DH5α competent cells (Tiangen, Beijing, China). The positive clones that ranged in size from 200–800 bp were tested by using vector primer T_7_/Sp_6_ and specific primer (AC)_10_/(AG)_10_/(AAG)_7_, respectively. In total, 369 clones were chosen to be sequenced with an ABI PRISM 3730XL DNA sequencer. The repeats were screened and counted with software Tandem Repeats Finder 4.0 [10]. We found 229 (62%) sequences containing microsatellite repeats. of which 59 were suitable for designing locus-specific primers using Primer 5.0 program [11].

Polymorphisms of all 59 microsatellite loci were assessed in 24 individuals of *Erigeron breviscapus* from five wild populations in Yunnan Province. The PCR reactions were performed in 15 μL volumes containing 50–100 ng genomic DNA, 0.6 μM of each primer, 7.5 μL 2 × Taq PCR Mastermix (Tiangen, Beijing, China; 0.1 U Taq Polymerase/μL, 0.5 mM dNTP each, 20 mM Tris–HCl (pH8.3), 100 mM KCl, 3 mM MgCl_2_). PCR amplifications were conducted under the following conditions: initial denaturation of 3 min at 94 °C followed by 32 cycles of 30 s at 94 °C, 30 s at the annealing temperature for each specific primer (optimized for each locus, Table 1), 72 °C for 1 min, and a final extension step at 72 °C for 7 min. The amplified products were separated on 6% denaturing polyacrylamide gel using a 20-bp ladder molecular size standard (Fermentas, Burlington, Ontario, Canada) by silver staining.

## 4. Conclusions

The 11 microsatellite markers developed in this study are the first set of microsatellite markers for *Erigeron breviscapus*. These polymorphic microsatellite markers will be used to investigate population genetic structure and genetic diversification of this species, as well as identify the cultivated varieties. These 11 polymorphic loci could also be useful for exploring population genetics and phylogenetics of congeneric species, especially those that are closely related to *Erigeron breviscapus*.

## Figures and Tables

**Table 1:** Characterization of 11 microsatellites isolated from *Erigeron breviscapus*.

Locus	GenBank Accession NO.	Repeat Motif	Primer Sequences(5′-3′)	Size Range (bp)	*Ta* (°C)	*A*	*H_O_*	*H_E_*
EB-2	HM173666	(AC)_7_-(AC)_9_-(ACC)_5_	F: CAAAAAGAAAACCACCCCC R: ACGCCGAAGGAGAAAGAG	144–170	58	6	0.708	0.612
EB-8	HM173667	(GT)_11_-(AG)_5_	F: CCACCAAAGTGCCAAATCC R: CAAAACCCTTACACCCTCCC	275–285	60	4	0.958	0.685 *
EB-10	HM173668	(AC)_13_(CT)_3_CAT(CT)_3_TT(CT)_2_	F: TCATTTACCCTTATCTCC R: GGTGTAAGAATTTTAGTGAG	100–110	57	5	0.542	0.732 *
EB-11	HM173669	(GT)_6_	F: AAGCGTGTACGTGTGTTC R: CCTTTTCATCTTCCAGTCTC	143–157	57	3	0.833	0.528 *
EB-17	HM173670	(TTG)_5_-(GTG)_6_-(TGG)_4_-(GT)_4_TT(GT)_9_TT(GT)_4_	F: CTAAAACATCATCTTCCAC R: ACTTATTTCCCCTTCCTC	195–225	53	4	0.417	0.426
EB-28	HM173671	(AG)_2_A(AG)_4_A(AG)_3_	F: AAGGAGGATGAGGGTGT R: GCAAGAGTGTTAGTGGG	135–145	62	3	0.917	0.582 *
EB-30	HM173672	(CT)_11_(TA)_7_	F: AGGCTACTTTGAAGGTTTCA R: AATCTAACCCACCCCTATG	220–266	54	7	0.250	0.786 *
EB-40	HM173673	(AC)_11_	F: GTAAACCAGCAGGCACT R: ATGGAGATGGAGGGATG	140–160	54	6	0.792	0.726
EB-47	HM173674	(TC)_12_-(CA)_9_TA(CA)_4_	F: AGGTATTTTCGGGTCAC R: AACTGCCACGTCAAGTA	170–184	54	4	0.792	0.533
EB-48	HM173675	(TTC)_5_	F: CCAGTCAGTGGGTAAAGTATG R: GAGTTTGTCCAAGAGAGGTG	180–190	58	2	0.417	0.337
EB-55	HM173676	(GA)_5_CA(GA)CA(GA)_2_	F: GAGATTATCGTGTTGCCG R: AGGACCCTGTTGAAAGTTAC	245–259	55	3	0.250	0.370

*T_a_*, PCR annealing temperature; *A*, number of alleles; *H_O_*, observed heterozygosity; *H_E_*, expected heterozygosity;

* Statistically significant deviation from Hardy-Weinberg equilibrium (*P* < 0.01).

**Table 2:** Congeneric species (*E. canadensis* and *E. altaicus*) amplification of 11 microsatellite loci isolated from *Erigeron breviscapus*.

Locus	*E. canadensis* (*n* = 4)	*E. altaicus* (*n* = 4)
EB-2	N	P(2)
EB-8	W	M
EB-10	N	N
EB-11	P(3)	M
EB-17	M	N
EB-28	M	M
EB-30	N	N
EB-40	N	P(2)
EB-47	M	P(2)
EB-48	M	P(2)
EB-55	N	M

N = no amplification; W = weak amplifications; M = monomorphic amplification; P = Polymorphic amplification (number of alleles)
